# Utility of evaluating symptom changes in the patient with patulous Eustachian tube through acoustic assessment: A case report

**DOI:** 10.1097/MD.0000000000043034

**Published:** 2025-06-27

**Authors:** Jin-Kook Lee, Cha Dong Yeo, Myoung-Hwan Ko, Gi-Wook Kim

**Affiliations:** a Department of Physical Medicine and Rehabilitation, Jeonbuk National University Medical School, Jeonju, Republic of Korea; b Department of Otorhinolaryngology-Head and Neck Surgery, Jeonbuk National University Medical School, Jeonju, Republic of Korea; c Research Institute of Clinical Medicine of Jeonbuk National University – Biomedical Research Institute of Jeonbuk National University Hospital, Jeonju, Republic of Korea.

**Keywords:** acoustic analysis, hypernasality, patulous Eustachian tube, therapeutic approaches for the treatment of middle ear disease

## Abstract

**Rationale:**

Patulous Eustachian tube (PET) refers to condition where the Eustachian tube remains abnormally open or fails to close adequately, leading to autophony, aural fullness, breathing autophony, tinnitus, and hypernasality. This case study reports the acoustic characteristics of a PET patient with hypernasality evaluate the changes in symptoms before and after conservative therapy, and demonstrate the utility of objective assessment through speech evaluation in addition to subjective symptom assessment.

**Patient concerns:**

The 40-year-old female presented to the otolaryngology outpatient clinic with complaints of aural fullness and autophony, tinnitus, hypernasality. Otoscopic examination revealed bilateral adhesive tympanic membranes. Despite bilateral ventilation tube insertion performed to address the persistent aural fullness, the patient continued to experience the same symptoms postoperatively.

**Diagnoses and Interventions:**

The patient began evaluation and treatment including speech, physical, and drug therapy were performed, for hypernasality, dyspnea, and swallowing problem with the Department of Rehabilitation Medicine. We assessed nasalance, vocal quality, and questionnaire both before and after the treatment. The acoustic data related to PET and the questionnaire results were then obtained.

**Outcomes:**

Significant improvements were observed in nasalance and vocal quality, questionnaire. Nasalance and jitter, shimmer metrics, questionnaire results gradually decreased. Notably, jitter and shimmer, questionnaire score, which were outside the normal range in the pretreatment assessment, returned to within normal limits following the conservative treatment.

**Lessons:**

Studies focused on PET with hypernasality changes in acoustic data and questionnaire are extremely rare. This case is significant in that not only was PET with hypernasality improved after conservative therapy, but also changes in nasalance, voice quality, and questionnaire were observed.

## 1. Introduction

The Eustachian tube (ET) prevents the backflow of sound or fluids from the nasopharynx.^[1]^ It also maintains consistent pressure when there is pressure difference between the middle ear and the nasopharynx.^[2]^ Patulous ET (PET) is dysfunction of the ET, characterized by its abnormally persistent opening or insufficient closure.^[3,4]^ Risk factors for PET include weight loss, pregnancy, medication, rhinopharyngeal irradiation, allergies, and gastroesophageal reflux.^[3,5]^ The incidence rate is 0.3% to 10%, occurring more frequently in women, and it is relatively rare disease that occurs in about 2% of patients with chronic otitis media.^[2,3]^ Symptoms of PET include voice autophony, aural fullness, breathing autophony, tinnitus, and nasalance problems.^[3]^ PET research was first introduced by Toynbee in the 1850s, with Schwartz reported the tympanic membrane movements in PET patients in 1864. Since then, several cases have been reported.^[2,6]^ However, there have been few studies on nasal voice among PET studies, in particular, there have been few studies on hypernasality.^[4]^ This study aims to report the acoustic characteristics of PET patients with hypernasality, evaluate the changes in symptoms before and after conservative therapy, and demonstrate the utility of objective assessment through speech evaluation in addition to subjective symptom assessment.

## 2. Case description

**Figure 1.**
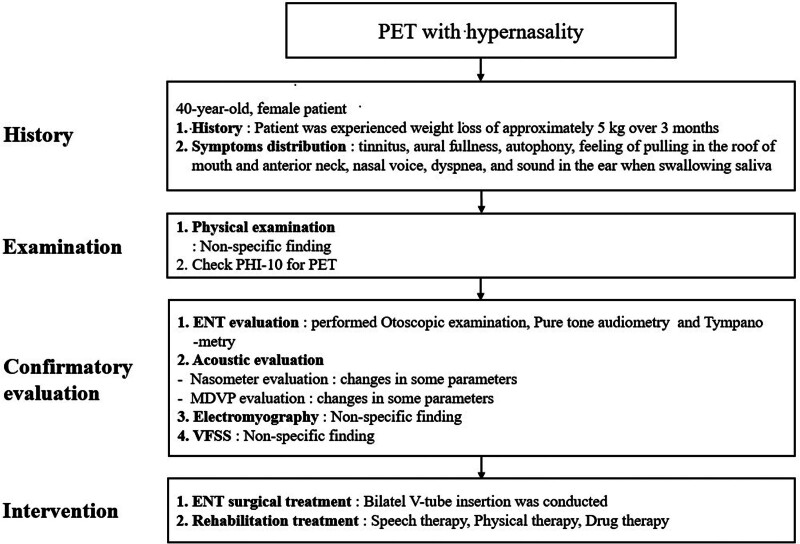
Flow chart of grading system for PET. PET = patulous Eustachian tube, PHI = patulous Eustachian tube handicap inventory, MDVP = multi-dimensional voice program.

**Figure 2.**
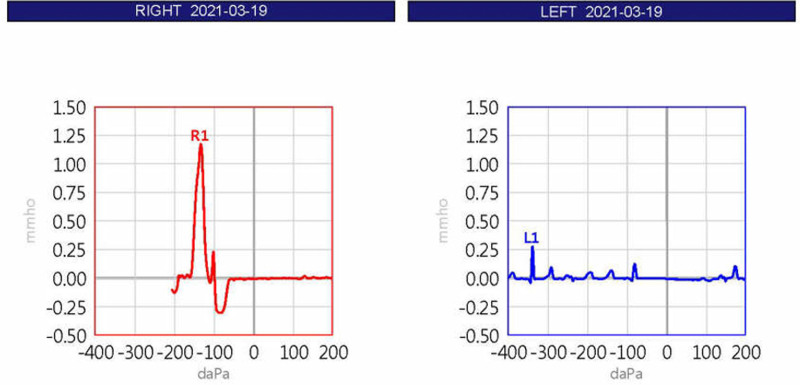
Tympanometry showed movement of the curve in sync with breathing.

The patient was a 40-year-old woman with a several-month history of aural fullness and autophony (Fig. 1). She presented to the otolaryngology outpatient clinic. Otoscopy revealed mildly retracted tympanic membranes in both ears, which moved with inspiration and expiration during the patient’s breathing. Pure tone audiometry showed no abnormalities, but tympanometry demonstrated movements of the tympanometric curve in sync with her breathing (Fig. 2). She had no previous history of chronic diseases and had experienced a weight loss of approximately 5 kg over the past 3 months. The patient was prescribed medication for the diagnosis of PET. However, despite medication, her symptoms of autophony, aural fullness, tinnitus, hypernasality, dyspnea and swallowing problem persisted, leading to bilateral ventilation tube insertion. Even after the procedure, the symptoms persisted. The patient began evaluation and treatment including speech, physical, and drug therapy were performed, for hypernasality, dyspnea and swallowing problem with the department of rehabilitation medicine.

We conducted physical examination. There were no other abnormal findings on physical examination. We checked the PHI (PET handicap inventory)-10, a modified version of the THI (tinnitus handicap inventory)-12, to assess the patient’s discomfort related to PET.^[7]^ Also we performed the Nasometer II 6450 (KayPentax Electrics, Lincoln Park, 2003) for nasalance measurements, which simultaneously inputs nasal and oral acoustic energies through 2 channels.^[8]^ Shure SM48 microphone was used for voice recording, and the patient’s voice was analyzed using the multi-dimensional voice program (MDVP) model 5105, KayPentax). The patient was asked to sustain the vowel/a/ for more than 5 s at a comfortable intensity and pitch, and to repeat this 3 times. And we performed electromyography, video-fluoroscopic swallowing study (VFSS), to confirm the neural damage. There were no abnormal findings on physical examination, electromyography, VFSS.

We applied PET intervention through conservative treatment and medication. In speech therapy, we utilized voice analysis and training instruments such as the nasometer and visi-pitch to provide visual feedback techniques apply.^[9,10]^ In visual feedback, the treatment task started with simple vowel production, then progressed to meaningless syllables consisting of oral consonants, and finally to words and sentences containing only oral consonants and vowels, excluding nasal consonants.^[11]^ In physical therapy, sternocleidomastoid muscle (SCM), scalene muscle stretching techniques were applied.^[12,13]^ In medication, muscle relaxants were prescribed.^[14]^ The intervention program included weekly sessions in the clinic with home exercises assigned, totaling 35 sessions.

**Figure 3.**
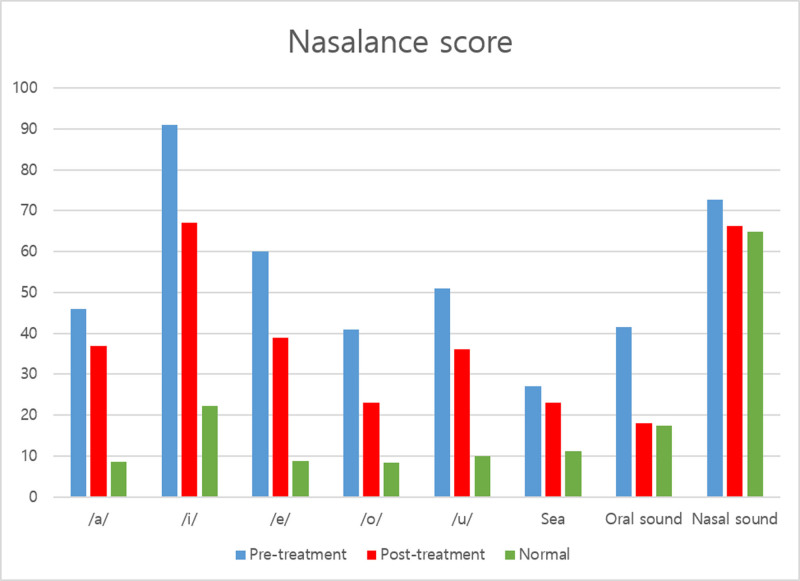
Nasalance score (vowel, sentence: sea passage, oral, nasal sound).

Vowels/a,i,e,o,u/ and nonnasal sentence (sea passage), oral sound, nasal sound were measured nasalance scores (%) and summarized (Table 1). Nasalance scores for vowels were from /a/ 46, /i/ 91, /e/ 60, /o/ 41, /u/ 51 to /a/ 37, /i/ 67, /e/ 39, /o/ 23, /u/ 36. For sentence scores were from 27 to 23, for oral sound from 41.5 to 18, for nasal sound from 72.7 to 66.2. After treatment nasalance scores showed decreased (Fig. 3). In MDVP, the acoustic analysis was performed on a stable 1-s segment of the sustained vowel /a/ for more than 5 s and summarized (Table 2). The jitter was changed from 1.797% to 0.443%, shimmer was from 4.159% to 2.557%, noise-to-harmonic ratio (NHR) was from 0.130 to 0.117. Also in PHI-10 score showed changes from 80 to 7. Since the patient complained of hypernasality while speech, we assessed the nasality and voice quality of speech. During the speech therapy, there was no change in medications, other interventions and there was no adverse effect such as more severe hypernasality or otolaryngologic diseases.

**Table 1:** Nasalance Scores (%) for vowels and sentence, oral, nasal sound.

Variable	Nasalance score (%)	
	Before treatment	After treatment
/a/	46	37
/i/	91	67
/e/	60	39
/o/	41	23
/u/	51	36
Sentence	27	23
Oral sound	41.5	18
Nasal sound	72.7	66.2

**Table 2:** Voice analysis parameters for vowel /a/.

Variable	Analysis parameter value		Normal
	Before treatment	After treatment	
Fo (Hz)	228.0	215.9	219.795 ± 17.896
Jitter (%)	1.797	0.443	0.816 ± 0.444
Shimmer (%)	4.159	2.557	2.973 ± 0.803
NHR	0.130	0.117	0.122 ± 0.016

## 3. Discussion and conclusion

The normal function of the ET is to prevent the transmission of infections from the nasopharynx to the middle ear and to maintain consistent pressure in the middle ear during activities like speaking or breathing.^[15]^ PET patients experience annoying symptoms such as voice autophony, aural fullness, and breathing autophony due to the persistently open ET.^[2,16]^

According to previous studies on the nasalance related PET. Suzuki reported that nasal voice can indicate PET severity.^[4]^ Ikeda reported that significant acoustic patency of nasal sounds in PET patients during speech, due to pressure differences between the nasopharyngeal and oropharyngeal spaces.^[7]^ Hassan reported that PET case with hypernasality following rapid weight loss.^[17]^

Research on PET with hypernasality rare, and there are no studies that have reported treatment effects using both objective and subjective evaluations along with questionnaires. Therefore, this study aims to report the acoustic characteristics of PET patients with hypernasality, evaluate the changes in symptoms before and after conservative therapy, and demonstrate the utility of objective assessment through speech evaluation in addition to subjective symptom assessment.

Our patient complained PET with hypernasality. After treatment, severe nasalance in vowels /a,i,u,e,o/ general decreased, with severity^[18]^ from moderate to mild except for vowels /i/,/e/. And compared to the before and after treatment, the greatest decrease showed in the front vowel /i,e/. In the before treatment, the nasalance for consonants in oral sounds was 41.5, but it decreased to 18. The nasalance score for nasal sounds also decreased from 72.7 to 66.2, which falls within the normal range.^[18]^ In the sentence evaluation, the before treatment nasalance indicated severe level of 27, but this decreased to moderate level of 23 in the after treatment, showing an overall reduction in nasalance. In addition, it was showed that the an objective nasalance assessment can be helpful judging symptom improvement in PET with hypernasality.

The ET is closed at rest, but opens during swallowing or yawning. The tensor veli palatini muscle (TVPM) and the levator veli palatini muscle (LVPM) contract simultaneously, allowing air to pass through the ET and equalizing the pressure in the middle ear with the atmosphere.^[19]^ The TVPM plays an essential role in opening the ET, and the synergy between the LVPM and TVPM is closely related to opening the ET.^[20–22]^ In addition, the LVPM is responsible for the main function of soft palate movement, which is related to nasal sound production.^[23]^ In particular, the TVPM is associated with high risk of middle ear disease.^[6]^

In this case, the before treatment showed high scores in nasalance and PHI-10 questionnaires, but the after treatment showed decrease in nasalance and PHI-10 questionnaire scores, indicating improvement in PET symptoms. These improvements suggest that an association between PET symptoms and TVPM function related to ET opening and LVPM function related to hypernasality.^[19,20,24–27]^

**Figure 4.**
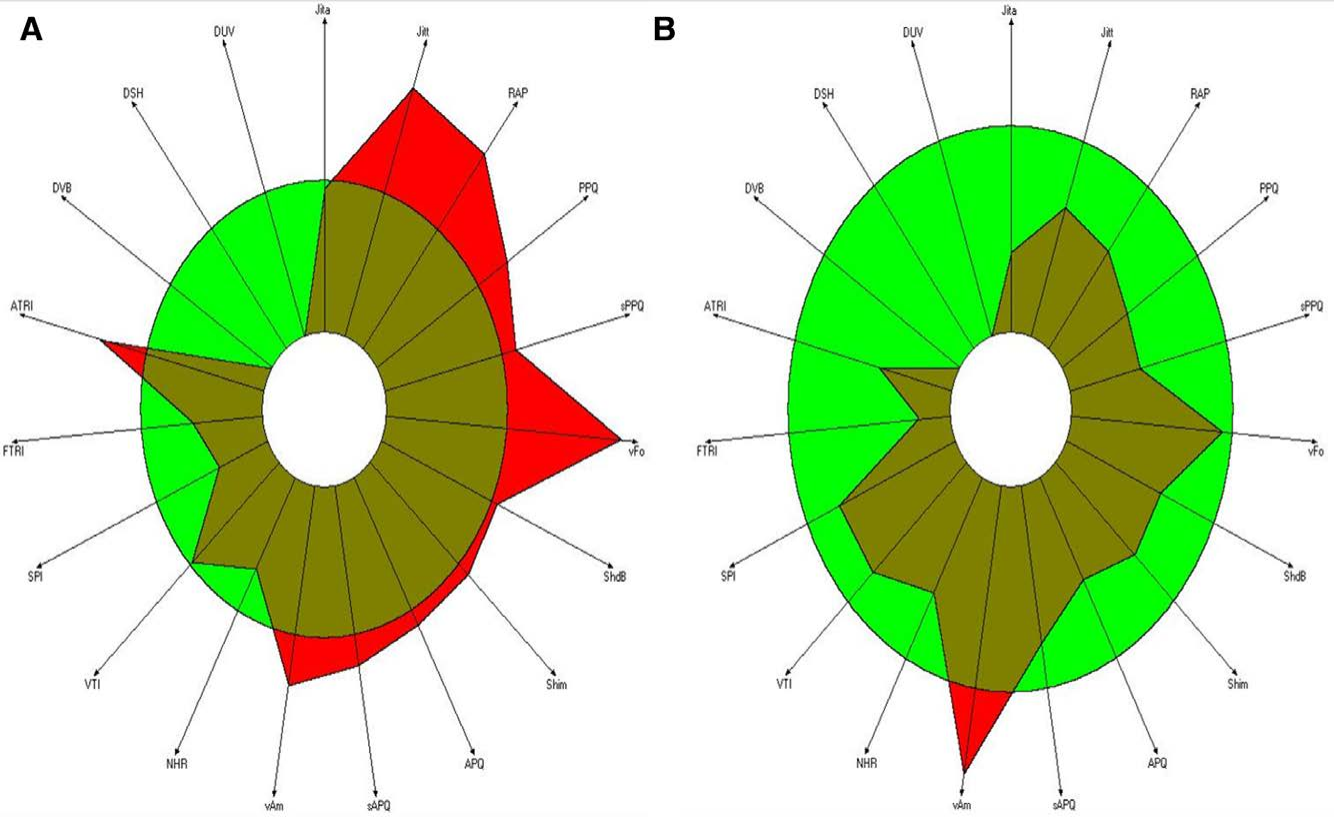
In MDVP on radial graph, the green ring represents the normal range, and the red area shows the subject’s voice condition. If the red area extends beyond the boundary of the circle, it indicates that the voice parameters are outside the normal range. (A) Before treatment. (B) After treatment. MDVP = multi-dimensional voice program.

MDVP analysis showed that fundamental frequency (Fo) was from 228 to 215.9 Hz. Jitter, which indicating pitch perturbation, decreased from 1.797% to 0.443%, jitter was out of the normal range, falling within the normal range in the posttreatment. Shimmer, which indicating amplitude perturbation, decreased from 4.159% to 2.557%, shimmer was out of the normal range, falling within the normal range. This is consistent with previous studies showing that the jitter and shimmer of hypernasal voice are significantly higher than those of the normal group (Table 2).^[28–31]^ NHR showed no significant difference, from 0.130 to 0.117 with values within the normal range. In the parameters representing voice quality, Fo and NHR were found to be within the normal range in both the before and after treatment. However the before treatment, relative average perturbation (RAP), pitch perturbation quotient (PPQ), smoothed pitch perturbation quotient (sPPQ), and fundamental-frequency variation (vFo), which represent the parameters related to the long and short-term fundamental-frequency information of the voice, were out of the normal range. Smooth amplitude perturbation quotient (sAPQ) and peak-amplitude variation (vAm), which represent the voice intensity of long and short-term sounds, and the ATRI (Amplitude Tremor Intensity Index) parameter, which represents the tremor of the voice, were out of the normal range. However, in the after treatment, only vAm was found to be out of the normal range. In addition, the number of parameters out of the normal range decreased in the after treatment compared to the before treatment (Fig. 4).

PHI-10, adapted from THI-12, is useful for evaluating PET. Despite other related questionnaires, PHI-10 is more suitable for PET patients with hypernasality and can indicate PET severity.^[7]^ Therefore, in this study, the before treatment showed high score of 80 points, but the after treatment showed low score of 7 points, which can be used as an indicator to help in the subjective evaluation of PET patients. This finding was consistent with previous studies.^[4,7]^

This case is meaningful in that not only changes in objective and subjective evaluations but also improvement of PET symptoms through conservative treatment. These findings could be an objective evidence to explain effectiveness of conservative therapy in treating PET with hypernasality. We report the rare case of changes in acoustic metrics and questionnaire related to PET with hypernasality and the efficacy of conservative interventions.

## Acknowledgments

The authors extend their appreciation to all members of the Department of Physical Medicine & Rehabilitation and Otorhinolaryngology-Head and Neck Surgery at Jeonbuk National University Hospital.

## Author contributions

**Conceptualization:** Jin-Kook Lee, Gi-Wook Kim.

**Data curation:** Jin-Kook Lee, Cha Dong Yeo.

**Formal analysis:** Jin-Kook Lee, Gi-Wook Kim.

**Funding acquisition:** Myoung-Hwan Ko.

**Investigation:** Jin-Kook Lee, Myoung-Hwan Ko, Gi-Wook Kim.

**Methodology:** Jin-Kook Lee, Cha Dong Yeo, Gi-Wook Kim.

**Resources:** Jin-Kook Lee, Cha Dong Yeo, Myoung-Hwan Ko.

**Software:** Jin-Kook Lee.

**Supervision:** Gi-Wook Kim.

**Validation:** Gi-Wook Kim.

**Visualization:** Jin-Kook Lee.

**Writing – original draft:** Jin-Kook Lee.

**Writing – review & editing:** Gi-Wook Kim.
